# Creutzfeldt-Jakob dementia

**DOI:** 10.1590/0100-3984.2014.0109

**Published:** 2015

**Authors:** Fabiano Reis, Ana Laura Gatti Palma, Ricardo Schwingel, Hélio Henrique Jorge Torres, Mariana Mari Oshima, Luciano Souza Queiroz, Fábio Rogério

**Affiliations:** 1 Universidade Estadual de Campinas (Unicamp), Campinas, SP, Brazil.

*Dear Editor*, 

A 72-year-old woman with rapidly progressive dementia, behavioral changes and apraxia of gait for seven months, extrapyramidal signs and diffuse myoclonus. Electroencephalography demonstrated periodic electric activity with high amplitude acute phase waves diffusely distributed over the cortex. The cerebrospinal fluid was normal. Magnetic resonance imaging (MRI) was performed (Figure 1).

**Figure 1:**
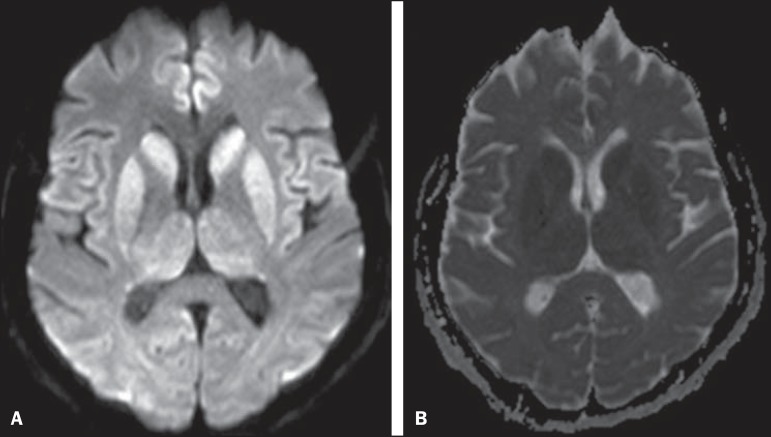
**A:** Axial magnetic resonance imaging of the skull demonstrating foci of hypersignal at diffusion-weighted sequences in the heads of the caudate nuclei, putamina, thalami and medial occipitotemporal gyri. **B:** At the ADC mapping, the low signal intensity in the same region confirms the diffusion restriction.

The association of clinical, radiological, electroencephalic or cerebrospinal fluid findings (presence of 14-3-3 brain protein in diseased patient for less than two years – absent in this case), allows for a probable diagnosis of Creutzfeldt-Jakob dementia (CJD). The differential diagnosis is made with other diseases associated with dementias, as follows: a) Alzheimer’s disease, that does not present with alterations observed at diffusion-weighted images; b) vascular dementia, with multiple infarcts, but with abnormalities at diffusion-weighted images only in cases of recent infarcts, and without diffuse cortical involvement; c) other encephalopathies that present alterations restricted to the cortex at diffusion-weighted images (such as MELAS – mitochondrial myopathy, encephalopathy, lactic acidosis and stroke-like episodes – a genetic metabolic disease occurring in younger patients); venous hypertensive encephalopathy and chronic herpes simplex encephalitis.

CJD is a subacute spongiform encephalopathy that presents with rapidly progressive dementia, and is the most frequent among rare prion diseases. Myoclonus, pyramidal, extrapyramidal and cerebellar signs are associated. Psychiatric symptoms are observed at the first six months; and progressive immobility, cortical blindness, dysphagia and mutism constitute late signs of the disease. Death generally occurs one year after the symptoms onset, since there is no therapy to prevent a fatal outcome^(1)^. The disease is subdivided into sporadic (85% of cases), familial, iatrogenic and a less common, recently described variant related to epidemic bovine spongiform encephalopathy^(2)^.

Like in other acute encephalopathies, the 14-3-3 brain protein may be present in the cerebrospinal fluid. Encephalography may demonstrate periodic activity composed of three-phase highfrequency waves over attenuated background activity.

At MRI, hyperintense signal is observed in the basal ganglia, putamen and later in the cerebral cortex on T2-weighted and FLAIR sequences^(1,3)^. Such alterations suggest CJD, more than any other dementia disorder^(4)^.

At early stages of the disease, however, conventional imaging studies may present normal results. Diffusion-weighted sequences may favor an early diagnosis, demonstrating abnormal hyperintense foci even before the appearance of electroencephalographic alterations, and should be performed in case of suspicion of CJD^(1,4,5)^. Diffusion restriction is observed, probably in association with the cytotoxic edema secondary to spongiform degeneration and to accumulation of abnormal cytoplasmic vacuoles. As the disease progresses, global atrophy is observed and, in general, this is the only alteration depicted at computed tomography^(1,4,6)^.

Histopathological analysis (Figure 2A) demonstrated spongiform alterations with variable intensity in the neuropil, markedly in the caudate nucleus, putamen, and in the region CA1 of the hippocampus, moderately in the frontal and temporal cortices, and slightly in the parietal and occipital cortices. Immunohistochemical analysis demonstrated gliosis (Figure 2B). Such results are compatible with a definite diagnosis of CJD. The lesions distribution, the absence of similar cases in the family, and the absence of a known infectious source are compatible com the sporadic presentation of the disease^(4,7)^. Such a diagnosis is relevant for controlling the transmission and to rule out treatable causes of dementia^(1>,8)^.

**Figure 2:**
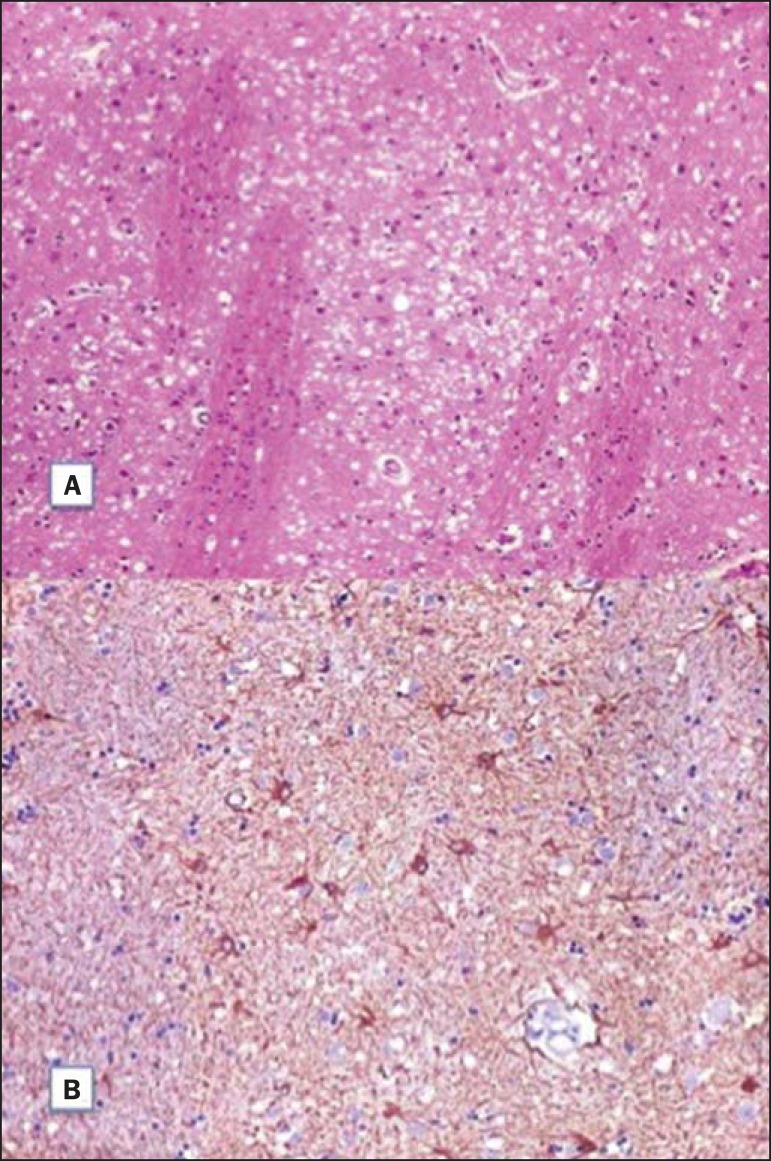
**A:** Section of the caudate nucleus demonstrating abundant small, optically empty vacuoles in the gray matter. The characteristic architecture of the caudate nucleus is highlighted by parallel white matter tracts through the gray matter (100× hematoxylin-eosin staining). **B:** GFAP immunohistochemistry demonstrating reactive astrocytes, a finding compatible with spongiform encephalopathy (400×).

## References

[1] Macfarlane RG, Wroe SJ, Collinge J (2007). Neuroimaging findings in human prion disease. J Neurol Neurosurg Psychiatry.

[2] Venneti S (2010). Prion diseases. Clin Lab Med.

[3] Meissner B, Kallenberg K, Sanchez-Juan P (2009). MRI lesion profiles in sporadic Creutzfeldt-Jakob
disease. Neurology.

[4] Ukisu R, Kushihashi T, Tanaka E (2006). Diffusion-weighted MR imaging of early-stage Creutzfeldt-Jakob
disease: typical and atypical manifestations. Radiographics.

[5] Shiga Y, Miyazawa K, Sato S (2004). Diffusion-weighted MRI abnormalities as an early diagnostic marker for
Creutzfeldt-Jakob disease. Neurology.

[6] Manners DN, Parchi P, Tonon C (2009). Pathologic correlates of diffusion MRI changes in Creutzfeldt-Jakob
disease. Neurology.

[7] Head MW (2013). Human prion diseases: molecular, cellular and population
biology. Neuropathology.

[8] Vitali P, Maccagnano E, Caverzasi E (2011). Diffusion-weighted MRI hyperintensity patterns differentiate CJD from
other rapid dementias. Neurology.

